# Numerosity estimation benefits from transsaccadic information integration

**DOI:** 10.1167/17.13.12

**Published:** 2017-11-01

**Authors:** Carolin Hübner, Alexander C. Schütz

**Affiliations:** AG Allgemeine und Biologische Psychologie, Philipps-Universität Marburg, Marburg, Germany

**Keywords:** saccades, numerosity, perceptual stability, transsaccadic integration, transsaccadic fusion, transsaccadic memory

## Abstract

Humans achieve a stable and homogeneous representation of their visual environment, although visual processing varies across the visual field. Here we investigated the circumstances under which peripheral and foveal information is integrated for numerosity estimation across saccades. We asked our participants to judge the number of black and white dots on a screen. Information was presented either in the periphery before a saccade, in the fovea after a saccade, or in both areas consecutively to measure transsaccadic integration. In contrast to previous findings, we found an underestimation of numerosity for foveal presentation and an overestimation for peripheral presentation. We used a maximum-likelihood model to predict accuracy and reliability in the transsaccadic condition based on peripheral and foveal values. We found near-optimal integration of peripheral and foveal information, consistently with previous findings about orientation integration. In three consecutive experiments, we disrupted object continuity between the peripheral and foveal presentations to probe the limits of transsaccadic integration. Even for global changes on our numerosity stimuli, no influence of object discontinuity was observed. Overall, our results suggest that transsaccadic integration is a robust mechanism that also works for complex visual features such as numerosity and is operative despite internal or external mismatches between foveal and peripheral information. Transsaccadic integration facilitates an accurate and reliable perception of our environment.

## Introduction

The majority of the human visual field conveys information with low visual resolution. Only a relatively small central part, the fovea, provides high-resolution visual information. Our visual system uses this architecture to locate potentially relevant objects in the periphery. Subsequently, the eyes move to project relevant objects onto the fovea and gain high-resolution information. With each of these eye movements, the position and resolution of objects on the retina changes, leading to the questions of how the brain achieves perceptual stability (for reviews, see Melcher & Colby, 2008; Mathôt & Theeuwes, 2011; Higgins & Rayner, 2015) and how pre- and postsaccadic information are combined. Recently, it has been shown that presaccadic peripheral information and postsaccadic foveal information are indeed integrated (Ganmor, Landy, & Simoncelli, 2015; Wolf & Schütz, 2015). Pre- and postsaccadic information were weighted according to their relative reliability, leading to statistically optimal integration according to the maximum-likelihood principle (Ernst & Bülthoff, 2004).

Although these studies present strong evidence for transsaccadic integration of information, they do not speak to a long-standing controversy in the study of transsaccadic perception, namely the level at which information is combined across saccades. Information could be combined at an early, image-based representation (transsaccadic fusion) or at a late representation (transsaccadic memory), when more abstract information has been extracted. In the 1980s, several studies refuted transsaccadic fusion (Jonides, Irwin, & Yantis, 1982, 1983; Bridgeman & Mayer, 1983; Irwin, Yantis, & Jonides, 1983; O’Regan & Lévy-Schoen, 1983) by presenting two stimuli that would yield a gestalt when combined in rapid succession. When the stimuli were presented during a fixation, participants fused the stimuli and easily recognized the gestalt. But when the stimuli were presented with a saccade in between, participants did not recognize the gestalt, suggesting that there was no fusion. However, a recent study provided evidence for transsaccadic fusion by reducing the duration and contrast of the postsaccadic stimulus, leading to fused percepts (Paeye, Collins, & Cavanagh, 2017). On the one hand, such a transsaccadic fusion mechanism might be very useful to aid the transfer of information across saccades. On the other hand, an image-based fusion might lead to distortions of visual perception when peripheral and foveal representations are incommensurate, for instance due to differences in resolution and sensitivity.

One example of a miscalibration between peripheral and foveal vision that might complicate transsaccadic integration is the perception of number in dot fields. Valsecchi, Toscani, and Gegenfurtner (2013) have shown that numerosity is underestimated in the periphery when compared to the fovea. Such a miscalibration is a challenge for transsaccadic integration and has to be compensated to achieve perceptual stability. Besides this inhomogeneity across the visual field, numerosity of dot fields is also interesting for the study of transsaccadic integration because these dot fields could be integrated on two distinct levels: on an image-based representation where, for instance, contrast information about each dot is combined across saccades, or on an abstract representation where global stimulus properties such as number are already extracted. Such an abstract representation should exist, since it has been shown that numerosity is a primary visual attribute that is analyzed independently from other visual attributes such as texture density (Burr & Ross, 2008; Anobile, Cicchini, & Burr, 2013; Cicchini, Anobile, & Burr, 2016). Interestingly, it has been suggested that the balance between numerosity and texture density differs between foveal and peripheral vision, due to differences in crowding (Anobile, Turi, Cicchini, & Burr, 2015). This could mean that peripheral information about dot fields is dominated by texture density and foveal information by numerosity. These complexities of numerosity perception make it questionable whether the visual system is nevertheless able to integrate information from the periphery and the fovea. Here we want to address this question and adapt the method of Wolf and Schütz (2015) to numerosity judgments.

In the first experiment, we compared perceptual performance in three conditions. In the foveal condition, information was presented to the participants solely in the fovea after a saccade. In the peripheral condition, as opposed to the foveal condition, solely peripheral information was shown before a saccade. To test whether there was an improvement in performance and compare this to the benchmark of maximum-likelihood integration (Ernst & Bülthoff, 2004), foveal and peripheral information was provided in the integration condition. In three further experiments, we studied which divergence of peripheral and foveal information can be tolerated by transsaccadic integration. This is an interesting question because it might help to elucidate which type of information—image based or abstract—is retained across a saccade and on which level transsaccadic integration operates. In Experiment 2, only local stimulus features, such as location and color of individual dots, were changed during the saccade. Previous research has shown that these local features are not necessarily represented and that local changes might be missed, especially under conditions of motion (Saiki & Holcombe, 2012). In Experiment 3, global stimulus features, such as the overall color of the dot field, were also changed during the saccade. Experiment 4 explicitly tested transsaccadic integration, as in Experiment 1, for the most extreme case of object discontinuity applied here.

## Methods

### Participants

Thirty-nine participants who were unaware of the purpose of our experiments and author CH participated in the first experiment (29 women, 11 men; mean age = 23 years, range = 19–33; all right-handed). We had to exclude the data for five participants because there were not enough valid trials, and of another participant because of a response bias leading to a strong deviation in the point of subjective equality. For all following experiments, we reinvited participants based on their performance in the first experiment. As we wanted to measure how disrupting object continuity impairs transsaccadic integration, reinvited participants’ data should indicate transsaccadic integration benefits in the form of showing better performance in the integration condition than in the single conditions. Thirteen of the reinvited participants (10 women, three men; mean age = 23 years) took part in the second experiment. Another 13 of the reinvited participants (nine women, four men; mean age = 22 years) took part in the third experiment. For the fourth experiment, 12 of the participants from Experiments 2 and 3 were tested (nine women, three men; mean age = 22 years). One of them showed an extreme decrease in performance compared to all other participants and was excluded from analysis. Observers were students of Marburg University and were reimbursed for participation. Experiments were in accordance with the principles of the Declaration of Helsinki and approved by the local ethics committee of the psychology department at Marburg University (proposal number 2015-35k). All observers gave informed consent and had normal or corrected-to-normal vision.

### Stimuli

Fixation stimuli were of a design that has been demonstrated by Thaler, Schütz, Goodale, & Gegenfurtner (2013) to be especially suitable for maintaining fixation on a screen. This combination of a bull’s-eye and crosshair had a diameter of 0.5° of visual angle and was used for fixation at the beginning of a trial as well as a target stimulus for saccade initiation in foveal trials. The color of the fixation stimulus was chosen randomly out of an array of colors of low contrast to avoid aftereffects. The color of the target stimulus was black to reduce variability of saccade latencies. Numerosity stimuli were circular windows filled with black and/or white dots on a gray background. The size of the circular window was kept constant, with a radius of 2.6° of visual angle. Dot positions were assigned randomly, with the constraint of having a minimal center-to-center distance of 0.15°. With a radius of 0.05°, the dots did not overlap. Depending on the trial, between 20 and 80 dots were presented. This corresponds to a dot density of 0.98–3.91 dots/°^2^. Masking stimuli in all experiments were spirals within a circular window with the same size and positions as the numerosity stimuli. The composing colors of the spirals were increments of black and white.

### Equipment

Stimuli were presented on a 91- × 51-cm back-projection setup with a PROPixx projector (VPixx Technologies, Saint-Bruno, Canada) and screen from Stewart Filmscreen (Torrance, CA). The screen had a resolution of 1,920 × 1,080 and a refresh rate of 120 Hz, with a viewing distance of 106 cm. Background luminance was 92 cd/m^2^ and the screen was calibrated to ensure a linear gamma correction. Luminance was at 3.3 cd/m^2^ for black pixels and 187 cd/m^2^ for white pixels. Eye movements were recorded with an EyeLink 1000 (SR Research Ltd., Ontario, Canada) with a sampling rate of 1000 Hz. Experimental software was written in MATLAB using the Psychophysics Toolbox (Brainard, 1997; Pelli, 1997). Participants responded using a standard keyboard.

### Procedure

**Figure 1:**
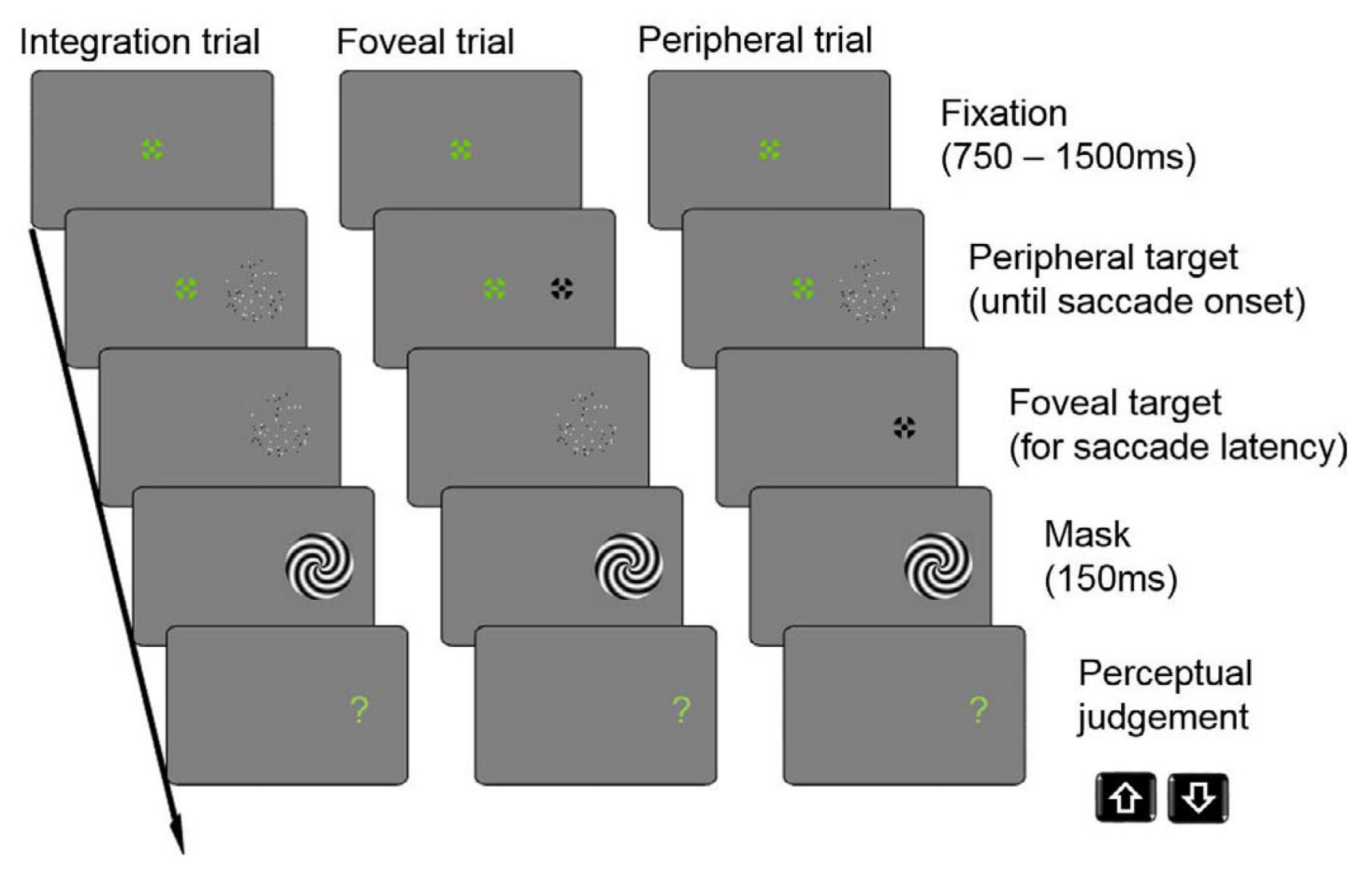
Trial procedure. Experiments 1 and 4 contained integration trials, foveal trials, and peripheral trials. In Experiments 2 and 3, only integration trials were tested. Every trial started with a fixation at the center of the screen. After a randomized interval, a saccade target appeared on the left or right at 12° eccentricity. The foveal target replaced this peripheral target as soon as a saccade was detected. In foveal trials, the peripheral target contained no numerosity information, whereas in peripheral trials, the foveal target contained no numerosity information. In integration trials, participants gained numerosity information from both parts of the visual field. A spiral mask appeared after a duration equal to the saccade latency beforehand, to limit the processing duration of the foveal target. After 150 ms, a question mark appeared to initiate the response of a key press of the up or down arrow on the keyboard.

The aim of Experiment 1 was to measure perceptual-discrimination performance for numerosity when different sources of visual information were provided. In integration trials (Figure 1), both sources of numerosity information—peripheral (before a saccade) and foveal (after a saccade)—were provided. Peripheral numerosity information was omitted for foveal trials. Therefore, the saccade target consisted of a black target stimulus which was replaced by a numerosity stimulus when a saccade was initiated. Conversely, the foveal numerosity information was dismissed for peripheral trials such that the numerosity stimulus was replaced by a black target stimulus as soon as the participant initiated a saccade.

In all trials, participants had to indicate whether the perceived number of dots was below or above the perceived mean numerosity of all previously presented stimuli in the experiment. A fixation stimulus at the screen center prompted participants to start the trial by fixating it and pressing the space bar simultaneously. After a random time between 0.75 and 1.5 s, a target appeared 12° left or right of the screen center. The fixation stimulus was removed after an additional 200 ms (overlap paradigm). Targets switched as soon as the EyeLink detected that the eye exceeded 1.6° with respect to the screen center. This guaranteed that the target was switched during the saccade, when vision is suppressed (for a review, see Ibbotson & Krekelberg, 2011).

A black-and-white spiral replaced the foveal target after its presentation duration to prevent any further visual processing of the numerosity stimulus. The mask was present for 150 ms. At the end of each trial, a question mark appeared at the target location to signal the participant that a response should be given. Participants could press either the up arrow key to indicate that the perceived numerosity was above the mean numerosity or the down arrow key to indicate that it was below the mean numerosity.

In every trial, the foveal target was displayed for the duration of the saccade latency of the participant of the specific trial. Thus, it was assured that participants saw the foveal target for the same duration as they saw the peripheral target. For instance, if it took 200 ms from target onset to target switch, the foveal target was presented for 200 ms as well. This guaranteed that observers were provided with roughly the same amount of peripheral and foveal information within each trial. To increase the likelihood that observers also had approximately the same viewing time across trials, they received feedback when the saccadic reaction time was too fast or too slow (target switch below 157.5 ms or above 257.5 ms). In these cases, a high or a low beeping sound was played but no visual feedback about the performance was provided. Observers were told to keep their eye-movement latency within the given time window. For the first 10 trials in each experiment, the experimenter remained with the participant to give advice and answer upcoming questions regarding the task. These 10 training trials in an experiment were omitted from analysis. At the end of Experiments 2, 3, and 4, questionnaires were filled out by the participants to reveal whether a change during a saccade reached conscious experience.

### Design

#### Experiment 1: Test for numerosity integration

In the first experiment, we measured transsaccadic integration of numerosity stimuli and compared perceptual performance to maximum-likelihood estimation. Integration and single trials (foveal or peripheral information only) were interleaved and pseudorandomized. The number of dots presented varied from 20 to 80 in eight steps (20, 30, 40, 44, 50, 56, 60, 70, and 80). Mean numerosity of the stimuli presented was 50 for all experiments. Psychometric functions per participant and conditions were sampled with nine data points based on at least an average of 10 observations. Each participant completed at least 486 trials in 45 min. Participants who successfully completed Experiment 1 and showed a better performance for integration trials than for single trials (see Results) could participate in Experiments 2, 3, and 4.

#### Experiment 2: Local disruption of object continuity

In Experiment 2 we studied how local changes in low-level stimulus properties affect integration performance. In the baseline condition, 50% of the dots were black and the other 50% white, and the numerosity stimulus stayed the same throughout the trial. In a comparison condition, the colors of the peripheral stimulus got swapped in the fovea, meaning that black dots turned white and white dots turned black. Please note that this manipulation affects only the local color of individual dots, not the summary statistics of the whole dot field. The same scheme was used for two additional conditions in which the proportion of black and white dots was 60%/40%. Here again, one condition involved no change between periphery and fovea and the other involved a color change in the fovea. To test for position change as well, one condition for the 50%/50% black and white stimuli involved a position change of the dots in the fovea. All five conditions were interleaved, and the experiment took around 90 min to complete. Participants completed at least 720 trials.

#### Experiment 3: Global disruption of object continuity

In this experiment, we made the changes between periphery and fovea more salient, such that they also affected the summary statistics of the dot field. Therefore, we used highly unbalanced proportions between black and white dots, namely 80%/20% and 100%/0%. The 50%/50% condition was additionally included for comparison. For all proportions of black and white, one condition included no change during the trial and another included a change of color during the saccade. Experiment 3 contained 864 trials and lasted for approximately 1 hr 45 min.

#### Experiment 4: Explicit test for integration in the 100%/0% condition

The purpose of this experiment was to test more explicitly whether participants still integrated peripheral and foveal numerosity information in the 100%/0% color-change condition. In this condition, object continuity was disturbed the most among our manipulations: All dots were black in the periphery and white in the fovea, or vice versa. As in our first experiment, we used single trials (peripheral and foveal trials) and integration trials to compare observed to predicted integration performance. Half of the integration trials contained no change in the numerosity stimulus; the other half contained a color change of the dots during the saccadic eye movement. The experiment contained 576 trials and lasted approximately 1 hr.

### Data and eye-movement analysis

Saccade onsets were detected offline using the Eye-Link 1000 algorithm. Saccade latencies were defined as the first saccadic frame with respect to target onset. To keep peripheral and foveal viewing time constant, trials with saccade latencies shorter than 100 ms or longer than 400 ms were excluded from further analysis. We excluded trials in which saccadic end points deviated from the target center by more than 2.5° of visual angle. This ensured that the target was fixated after the saccade and until the target disappeared. Taking together excluded trials for saccade latency and saccade end point, participants’ mean number of outliers was 8.5% ± 9.0% (range = 1.6%–49.1%) of trials. In Experiment 1 we excluded five participants who had too many invalid trials, such that the mean number of data points per fit of a psychometric function was less than 10. One participant was excluded for being more than 30% away from the true mean numerosity estimate, such that a sufficiently valid fit of a psychometric function could not be guaranteed.

Perceptual choices were converted into proportion of up-arrow responses for every stimulus numerosity, and a cumulative Gaussian was fitted to the data using psignifit 4.0 (Schütt, Harmeling, Macke, & Wichmann, 2015). The point of subjective equality (PSE) was estimated as the numerosity value corresponding to 50% up-arrow responses. Just-noticeable differences (JNDs) were defined as the standard deviation of the cumulative Gaussian. To test whether perceptual integration of numerosity is optimal according to the maximum-likelihood estimation model (for a review, see Ernst & Bülthoff, 2004), predicted JNDs for integration were calculated using

(1) $${\mathrm{JND}}_{\mathrm{int}\_\mathrm{pred}}=\sqrt{\frac{1}{{\mathrm{rel}}_{\mathrm{int}\_\mathrm{pred}}}}.$$

The predicted reliability of the integrated percept *rel_int_pred_* should be the sum of the individual reliabilities for foveal and peripheral presentation, if independence between cues is given:

(2) $${\mathrm{rel}}_{\mathrm{int}\_\mathrm{pred}}={\mathrm{rel}}_{\mathrm{per}}+{\mathrm{rel}}_{\mathrm{fov}}.$$

Reliabilities can be calculated given the JND for a participant and condition:

(3) $$\mathrm{rel}=\frac{1}{{\mathrm{JND}}^{2}}.$$

With the reliabilities at hand, the optimal peripheral weighting can be calculated by

(4) $$w_{\mathrm{per}}=\frac{{\mathrm{rel}}_{\mathrm{per}}}{rel_{\mathrm{per}}+{\mathrm{rel}}_{\mathrm{fov}}}.$$

Results were compared using one-way repeated-measures ANOVAs and post hoc *t* tests. If not noted otherwise, all *t* tests were two-tailed and *p* values were compared against a Bonferroni-corrected *α* of 0.05.

An important prerequisite for being able to compare performance in peripheral, foveal, and integration conditions in Experiments 1 and 4 is similar saccade latencies, because presentation durations were locked to saccade onsets. In Experiment 1 we found that mean saccade latencies were slightly longer for the foveal condition (219.63 ± 30.89 ms) compared to the peripheral (202 ± 25.37 ms), *t*(33) = 5.66, *p* < 0.001, and integration conditions, (203.05 ± 26.11 ms), *t*(33) = 5.78, *p* < 0.001. The same applies for Experiment 4: Foveal mean saccade latency (212.97 ± 19.45 ms) was significantly different from peripheral mean saccade latency (191.40 ± 18.05 ms), *t*(10) = 4.29, *p* = 0.002, from the integration no-change condition (189.72 ± 16.32 ms), *t*(10) = 5.94, *p* < 0.001, and from the integration with-change condition (189.19 ± 16.11 ms), *t*(10) = 5.32, *p* < 0.001. This means that the presentation time of the foveal target was on average slightly longer in the foveal condition than in the integration conditions. This might lead to an overestimation of foveal reliability, which would result in an overestimation of predicted reliability and an overestimation of foveal weights in integration conditions. However, the duration differences were only in the order of one to two monitor frames and therefore should have only small influences on perceptual performance.

## Results

### Experiment 1: Test for numerosity integration

The aim of the first experiment was to study whether participants used information from both parts of the visual field to estimate numerosity optimally. First, we analyzed whether participants were more accurate in integration trials, given both peripheral and foveal numerosity information, than in single trials, given foveal or peripheral information only. Accuracy is represented in the mean of the psychometric function— that is, the PSE—when more- and less-accurate responses are balanced. Second, we analyzed whether the precision of numerosity discrimination increased with combination of peripheral and foveal information. The precision is represented in the standard deviation of the psychometric function—that is, the JND. Finally, we compared the observed JNDs in the integration trials to JND values predicted by a maximum-likelihood estimation model (Ernst & Banks, 2002). These predicted JND values represent a benchmark for optimal transsaccadic information integration.

**Figure 2:**
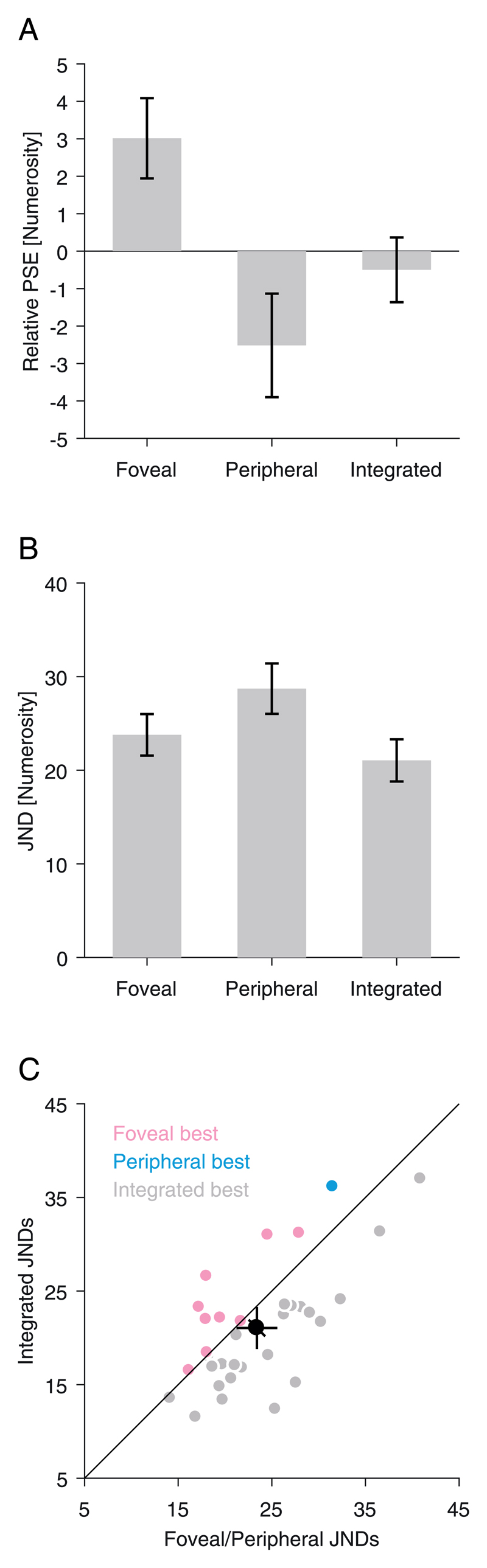
Relative values for point of subjective equality and values for just-noticeable difference (Experiment 1). (A) Normalized values for point of subjective equality with their 95% confidence intervals as error bars. Numerosity presented in the fovea only was slightly underestimated, whereas numerosity presented in the periphery only was slightly overestimated. Given both inputs, the point of subjective equality was closest to the true mean numerosity of the stimuli. (B) Comparison of mean values for just-noticeable difference over participants in the two single conditions and the integration condition, with 95% confidence intervals as error bars. Performance was lowest when only peripheral numerosity information was given, highest when foveal and peripheral information was provided, and in between when only foveal information was given. (C) Just-noticeable difference in integration condition as a function of the best single condition (peripheral or foveal) for every participant. Most participants were best in the integration condition (gray circles), whereas data points above the identity line were better in one of the single conditions (colored circles).

Obtaining PSEs (Figure 2A) from the psychometric functions per participant over all conditions revealed that participants in Experiment 1 rated the true mean numerosity (50) to be close to their perceived mean numerosity (49.9 ± 5.5), *t*(33) = −0.10, *p* = 0.925—not significantly different from 50). The PSE for the foveal condition was at 52.7 ± 6.8 (*M* ± *SD*)—significantly different from the true mean numerosity, *t*(33) = 2.29, *p* = 0.029. This means that participants perceived a higher numerosity at the center of the distribution and therefore underestimated numerosity in the foveal condition.^1^ The PSE for peripheral trials was at 47.21 ± 6.99, *t*(33)= −2.39, *p* = 0.023—significantly different from 50—showing an overestimation of numerosity in the periphery. The PSE in integration trials was not significantly different from the true mean numerosity (49.16 ± 5.46), *t*(33) = −0.90, *p* = 0.375—not significantly different from 50. Comparing the PSEs for the different conditions revealed significant differences between foveal and peripheral conditions, *t*(33) = 4.85, *p* < 0.001, and between foveal and integration conditions, *t*(33) = 5.21, *p* < 0.001. A difference close to significance was found for the peripheral and integration conditions, *t*(33) = −2.01, *p* = 0.053.

**Figure 3:**
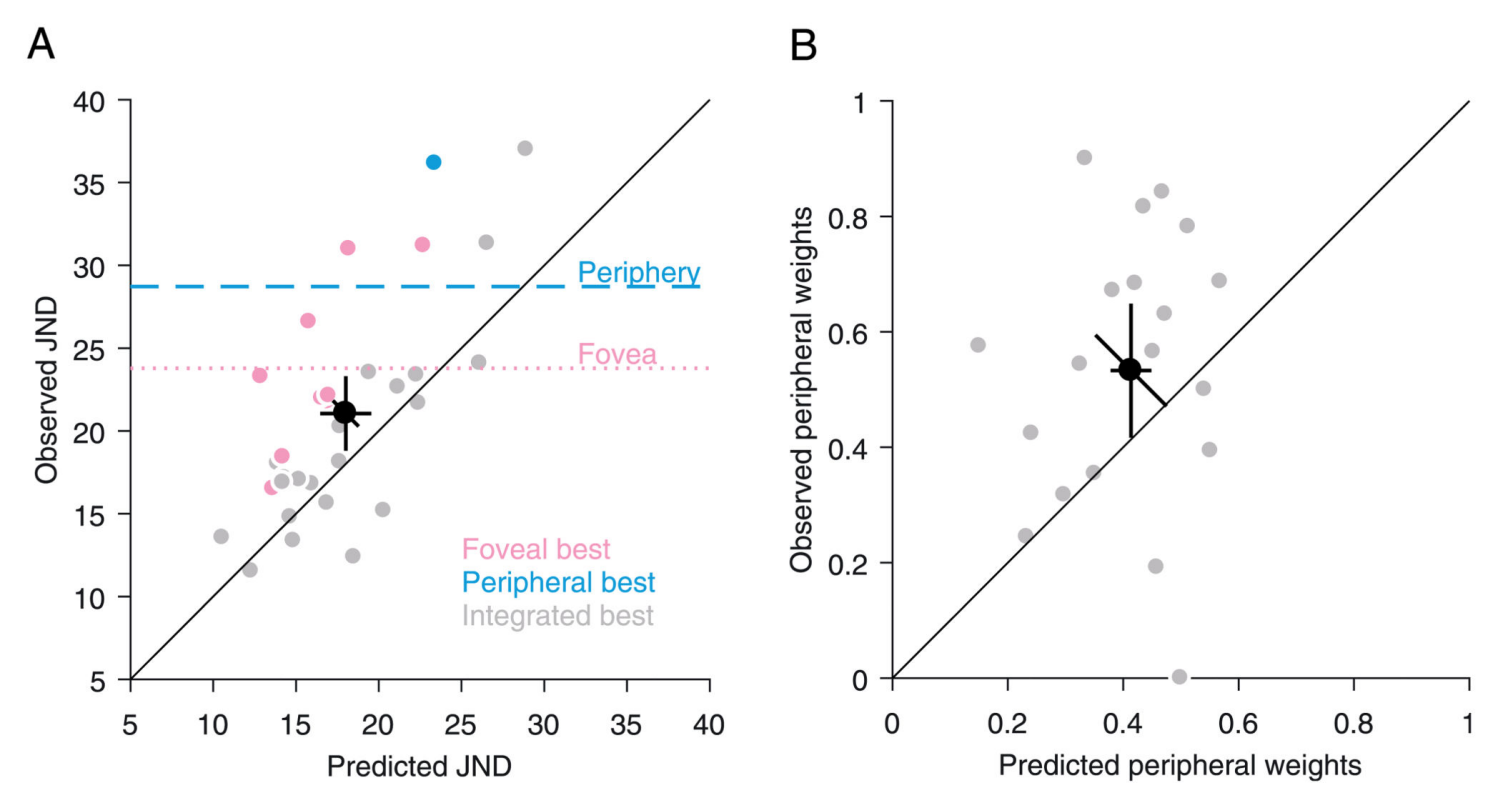
Optimality of integration and peripheral weighting (Experiment 1). (A) Individual data (gray and colored circles) and mean (black filled circle) comparing predicted values for just-noticeable difference (JND) for integration to observed JND values of the integration condition. Most individual data gather along the identity line indicating optimal transsaccadic integration. The mean over all participants indicates slightly worse performance than predicted, but close to optimal integration behavior. Horizontal lines depict the mean JND of the foveal and peripheral conditions. Both are above the mean JND of the integration condition. (B) Comparison of predicted peripheral weights and observed peripheral weights based on individual values for JND and point of subjective equality. Individual data are depicted as gray circles, and their mean as filled black circle, with error bars denoting 95% confidence intervals. The diagonal error bar marks the error of the differences between observed and predicted values, and has to be compared to the identity line (solid). Participants are shown to have relied on the peripheral information slightly more than predicted.

For the JNDs we found significant differences between all conditions (Figure 2B). Participants were significantly better at discriminating numerosity given foveal information only compared to peripheral information only (foveal: 23.79 ± 6.36; peripheral: 28.72 ± 7.72), *t*(33) = −4.64, *p* < 0.001. However, given both foveal and peripheral information in the integration condition (21.05 ± 6.45), participants were significantly better than in the foveal condition, *t*(33) = 3.13, *p* = 0.002 (one-sided), and the peripheral condition, *t*(33) = 7.36, *p* < 0.001 (one-sided). Figure 2C shows the comparison of individual JND values for integration and the best of foveal or peripheral conditions. The result confirms the finding that performance was generally better in the integration condition, *t*(33) = 2.71, *p* = 0.005 (one-sided). Only 11 out of 34 participants were worse in the integration condition than in the foveal or peripheral conditions. According to the maximum-likelihood estimation model, integration should be optimal when the predicted JND from the model equals the observed JND of the participant (Equations 1 and2). Figure 3A depicts this comparison and reveals a close-to-optimal integration for numerosity (observed JND: 21.05 ± 6.45; predicted JND: 18.01 ± 4.43), *t*(33) = −3.98, *p* < 0.001.

The differences between peripheral and foveal PSEs indicate a miscalibration of perceived numerosity across the visual field. Inappropriate weighting of miscalibrated signals could lead to a reduction in precision compared to the optimal predictions. To test whether peripheral and foveal information were appropriately weighted, we calculated the predicted peripheral weights (Equation 4) and compared them to the observed peripheral weights for the PSEs. For this comparison, we could only use data from participants whose PSE in the integration condition was in between their PSEs for the two single conditions (19 of 34 participants). Interestingly, the pattern (Figure 3B) indicated a higher weighting for peripheral information than predicted (observed: 0.53 ± 0.24; predicted: 0.41 ± 0.12), *t*(18) = −2.17, *p* = 0.044.

For our first experiment, participants accurately identified the true mean numerosity as their mean PSE (over all conditions). Even though numerosity perception differed significantly for peripheral and foveal vision, participants showed more accurate perception when both inputs were provided in the integration condition. The finding that the relation between the observed and predicted discrimination performance was close to the optimality line (Figure 3A) is additional evidence that numerosity information before and after a saccade is integrated almost optimally. Furthermore, participants’ discrimination performance in the integration condition was significantly better than in their best single condition (foveal or peripheral), as shown in Figure 2C.

### Experiment 2: Local disruption of object continuity

In our second experiment, we wanted to test whether integration performance is affected by disrupting object continuity. Therefore, we compared integration performance from a baseline condition to that of a change condition. The first baseline condition was a replication of the integration condition of the previous experiment. The stimuli consisted of 50%/50% black and white dots, and stayed the same throughout a trial. The corresponding change conditions differed in the way that either the color of the dots was exchanged (50%/50% color-change condition) or the dot positions were changed (50%/50% position-change condition) during the saccade. To make the color changes during a saccade more salient, a 60%/40% black-and-white-dots baseline condition was introduced, accompanied by a 60%/40% color-change condition.

**Figure 4:**
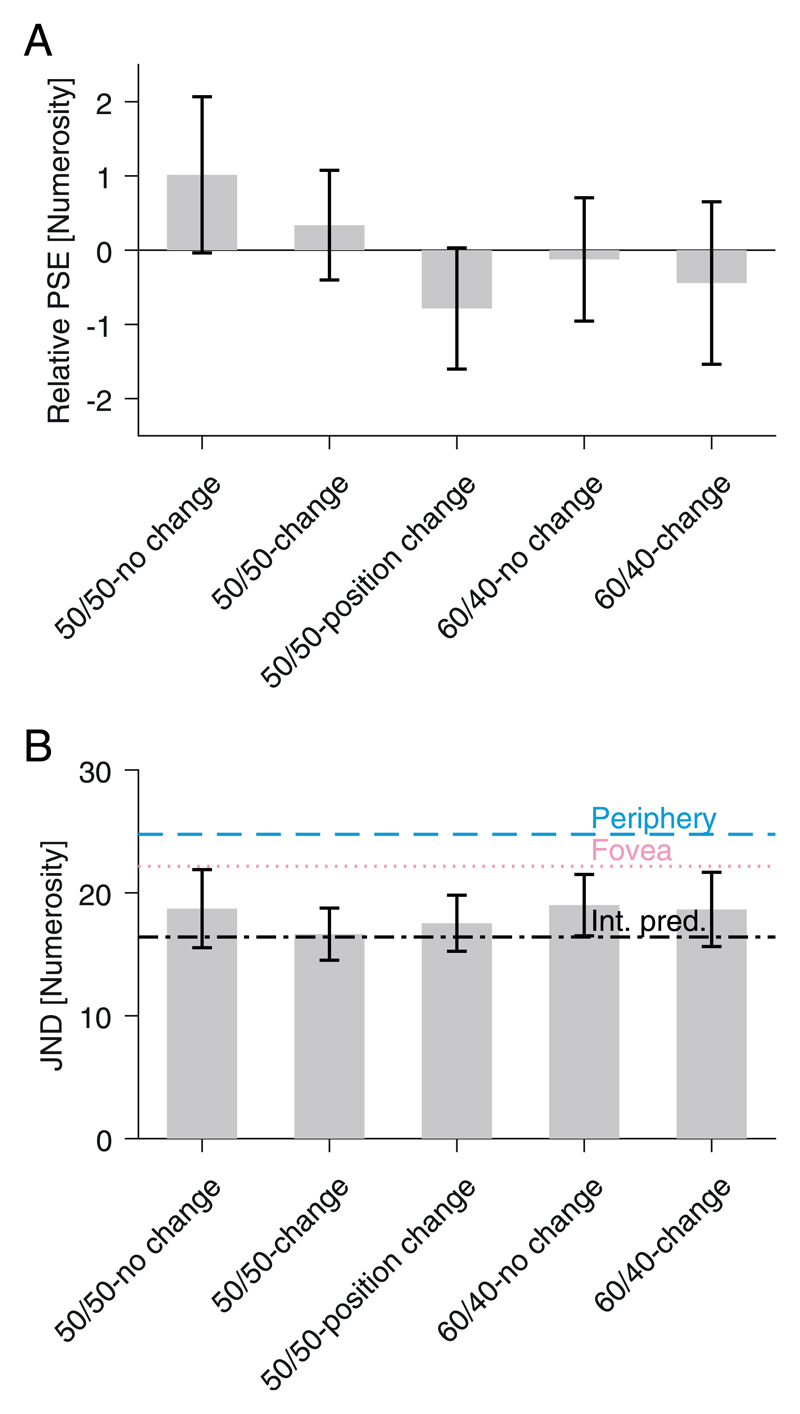
Values for point of subjective equality and just-noticeable difference (Experiment 2). (A) Relative values for point of subjective equality for all five conditions of the second experiment, with 95% confidence intervals as error bars. Numerosity was slightly overestimated in the 50%/50% position-change condition compared to its baseline condition (no change). (B) Mean values for just-noticeable difference over all conditions, with 95% confidence intervals as error bars. Performance did not depend on the object continuity manipulation. Horizontal lines show the mean values for just-noticeable difference for foveal, peripheral, and integration-predicted conditions from the first experiment.

We first compared the PSEs between the five different conditions for integration trials (Figure 4A). Mean PSEs over all conditions were slightly above but not significantly different from the true mean numerosity (51.70 ± 5.09), *t*(12) = 1.20, *p* = 0.252—not significantly different from 50. A one-way ANOVA indicated a significant difference between the conditions, *F*(4, 60) = 2.78, *p* = 0.035. Post hoc *t* tests between the baseline and change condition pairings revealed that participants slightly overestimated numerosity when the positions of the dots changed during a saccade compared to the no-change condition, *t*(12) = 3.04, *p* = 0.010. The average JND across all conditions in Experiment 2 (18.11 ± 4.34) was below the averaged JND values for those participants in the single conditions of Experiment 1—foveal (22.17 ± 4.17), *t*(12) = −4.07, *p* = 0.002; peripheral (24.76 ± 5.55), *t*(12) = −4.36, *p* < 0.001—and close to the average predicted JND for those participants in Experiment 1 (16.41 ± 3.16), *t*(12) = 1.80, *p* = 0.097. The ANOVA performed on the JNDs from Experiment 2 indicated no significant effect among the five conditions, *F*(4, 60) = 0.67, *p* = 0.618. An additional Bayes-factor analysis (for a review, see Jarosz & Wiley, 2014) supports the null hypothesis moderately (BF_01_ = 7.09).

The results of Experiment 2 are in favor of the hypothesis that the perisaccadic stimulus changes did not affect transsaccadic integration of numerosity. The finding that the JND values do not differ significantly between the baseline and change conditions supports this conclusion. Furthermore, the JNDs in all integration conditions of Experiment 2 were below the JNDs of the single conditions of Experiment 1, suggesting that participants integrated despite the intrasaccadic changes.

### Experiment 3: Global disruption of object continuity

Manipulating object continuity in the previous experiment did not affect transsaccadic integration. However, all of these manipulations affected local stimulus features, such as the color or location of individual dots, and left global stimulus features, such as the overall color, largely unaffected. Previous research has shown that changes in local features can go unnoticed easily under conditions of motion (Saiki & Holcombe, 2012) and that even the assignment of individual dots to one of two surfaces is limited (Schütz, 2012). In a similar way, such local changes might be overlooked during saccades and therefore leave integration performance unaffected. In this experiment, we challenged transsaccadic integration with changes in global stimulus features that should not be overlooked as easily: Proportions of black and white dots were chosen to be 80%/20% for one pair of conditions (baseline and color change) and 100%/0% for another pairing. As a result, the overall brightness of the dot field changes in the color-change conditions. The 50%/50% black-and-white proportion was included again for comparison.

**Figure 5:**
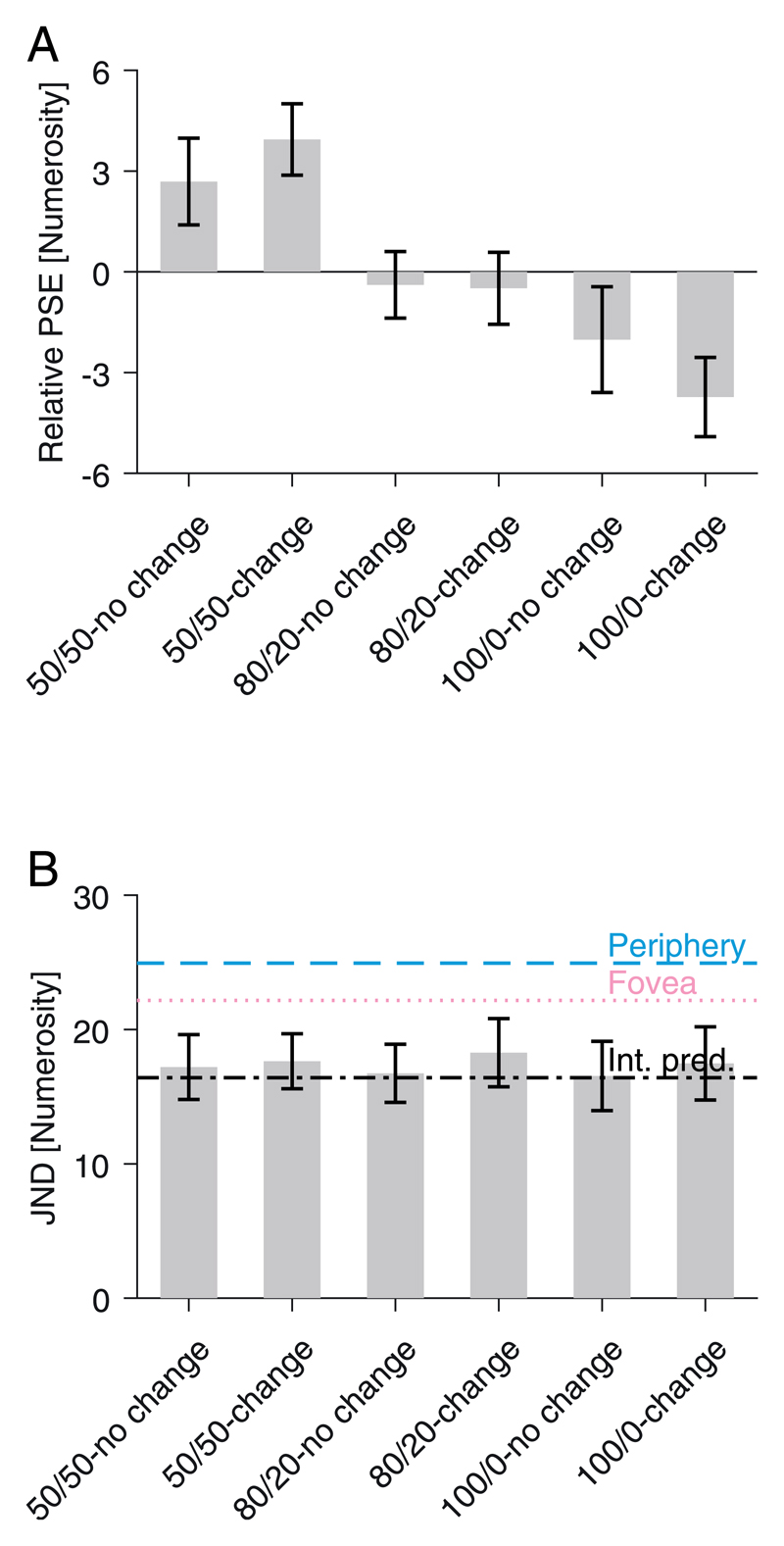
Values for point of subjective equality and just-noticeable difference (Experiment 3). (A) Relative mean values for point of subjective equality for all three pairs of baseline and color-change condition, with 95% confidence intervals as error bars. The degree of underestimation seems to shrink with an increased imbalance of black and white dots. (B) Mean values for just-noticeable difference over all conditions, with 95% confidence intervals as error bars. Discrimination performance was not affected by the object continuity manipulation. Horizontal lines show the mean values for just-noticeable difference for foveal, peripheral, and integration-predicted conditions from the first experiment.

As in the previous experiments, we first compared the PSEs between the six different conditions for integration trials (Figure 5A). Mean PSE over all conditions was not significantly different from the true mean numerosity (51.61 ± 4.58), *t*(12) = 1.27, *p* = 0.229—not significantly different from 50. The *t* tests between the baseline and change pairings revealed that participants overestimated numerosity more, or deviated more strongly from the true mean numerosity, in the 100%/0% color-change condition (47.69 ± 4.28) than in the 100%/0% no-change condition (49.40 ± 5.21), *t*(12) = 3.66, *p* = 0.003. Conversely, participants underestimated numerosity slightly more in the 50%/50% change condition (55.36 ± 5.41) compared to its baseline condition (54.11 ± 6.23), *t*(12) = −2.33, *p* = 0.038.

Participants’ average JND across all conditions in Experiment 3 (17.31 ± 3.92) was below the JNDs of those participants in the single conditions of Experiment 1—foveal (22.17 ± 4.40), *t*(12) = −3.38, *p* = 0.006; peripheral (24.94 ± 6.48), *t*(12) = −4.14, *p* = 0.001—and in the range of the average predicted JND of those participants in Experiment 1 (16.42 ± 3.50), *t*(12) = 0.75, *p* = 0.468. An ANOVA of the JND values from Experiment 3 indicated no significant difference overall, *F*(5, 72) = 0.32, *p* = 0.898. A subsequent Bayes-factor analysis supports the null hypothesis strongly (BF_01_ = 15.21).

The results of Experiment 3 show that even global disruptions of object continuity in terms of brightness or contrast polarity did not impair discrimination performance. The findings suggest that participants were still integrating peripheral and foveal information for all conditions. PSE values reveal that different proportions of black and white dots seem to influence numerosity estimation. The more unbalanced the proportions were, the more participants overestimated numerosities. However, these tendencies were not affected by color changes during a saccade.

### Experiment 4: Explicit test for integration in the 100%/0% condition

Showing that JNDs in Experiments 2 and 3 are close to predicted JNDs in Experiment 1 and do not differ among the different conditions does not fully prove that participants actually integrated the stimuli transsaccadically. Since participants were reinvited to Experiments 2 and 3, a training effect could also be the cause of the good performance found in these experiments. To rule out this possibility, we reapplied the design of Experiment 1 to explicitly compare performance in single trials (foveal or peripheral information only) to performance in integration trials (both provided) for a condition with disrupted object continuity. Among the different manipulations of Experiment 2 and 3, we chose the ratio of 100%/0% black and white dots, as it implies the most salient change when the colors get swapped. The aim of Experiment 4 was to compare integration performance for these stimuli when nothing changes during the saccade, as well as when the color changes during the saccade.

**Figure 6:**
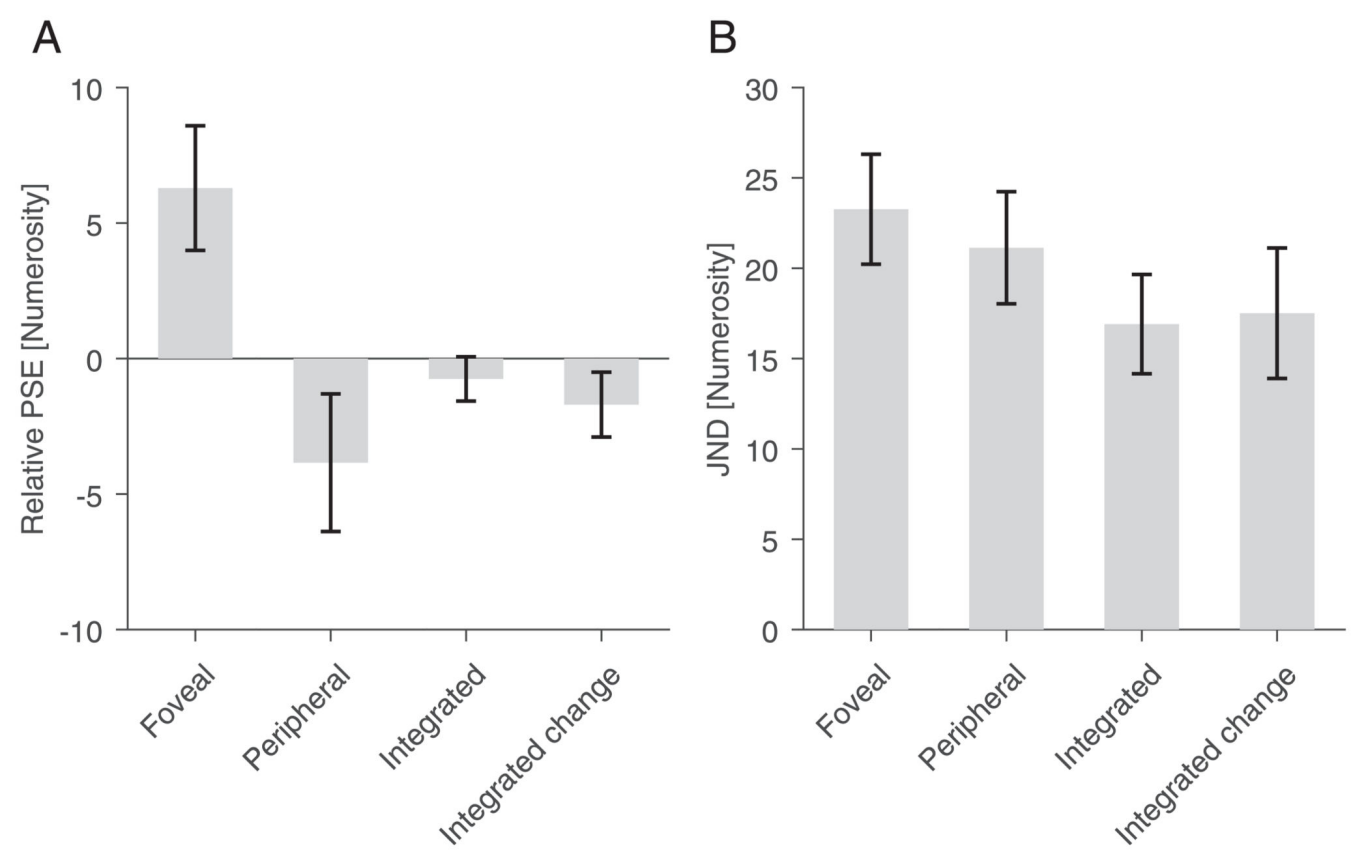
Values for point of subjective equality and just-noticeable difference (Experiment 4). (A) Relative mean values for point of subjective equality for the two single and the two integration conditions, with 95% confidence intervals as error bars. Numerosity was underestimated in the foveal condition, overestimated in the peripheral condition, and rather accurate in both integration conditions. (B) Mean values for just-noticeable difference over all conditions, with 95% confidence intervals as error bars. While the two single conditions and two integration conditions do not differ significantly within each pair, each condition of one pair differs significantly from each condition of the other.

Participants’ averaged PSEs over all conditions were again close to the true mean numerosity (52.48 ± 4.85), *t*(10) = 1.69, *p* = 0.121—not significantly different from 50. Averaged PSEs for the four individual conditions (Figure 6A) show that numerosity was underestimated in the foveal condition (58.66 ± 6.30), *t*(10) = 4.56, *p* = 0.001—significantly different from 50—while it was rather accurate for the peripheral condition (48.53 ± 6.80), *t*(10) = −0.72, *p* = 0.491—not significantly different from 50. The average PSE was also accurate in both the integration condition without a change (51.62 ± 4.77), *t*(10) = 1.13, *p* = 0.285—not significantly different from 50—and the integration condition with color change (50.68 ± 4.27), *t*(10) = 0.52, *p* = 0.612— not significantly different from 50. Our *t* tests between the PSEs in all four conditions revealed a significant difference between the foveal and peripheral conditions, *t*(10) = 4.87, *p* < 0.001, and between the foveal condition and the two integration conditions: foveal and integrated, *t*(10) = 6.75, *p* < 0.001; foveal and integrated with change, *t*(10) = 6.28, *p* < 0.001. The differences between peripheral and integration conditions only reached significance for the no-change condition: peripheral and integrated, *t*(10) = −2.31, *p* = 0.043; peripheral and integrated with change, *t*(10) = −1.57, *p* = 0.148. The PSEs of the two integration conditions did not differ significantly, *t*(10) = 1.41, *p* = 0.190.

An ANOVA of the JNDs over the four conditions indicated a significant difference between them, *F*(43) = 4.59, *p* = 0.008. Subsequent *t* tests revealed significant differences between the foveal (23.27 ± 4.53) and integration no-change conditions (16.91 ± 4.09), *t*(10) = 4.08, *p* = 0.001 (one-sided) and between the foveal and integration with-change conditions (17.51 ± 5.37), *t*(10) = 3.21, *p* = 0.005 (one-sided). The mean JND in the peripheral condition (21.13 ± 4.62) was also significantly different from those in the integration condition without change, *t*(10) = 3.28, *p* = 0.004 (one-sided), and with change, *t*(10) = 4.03, *p* = 0.001 (one-sided). Different from the results in the first experiment, there was no difference between peripheral and foveal discrimination performance, *t*(10) = 1.35, *p* = 0.208.

**Figure 7:**
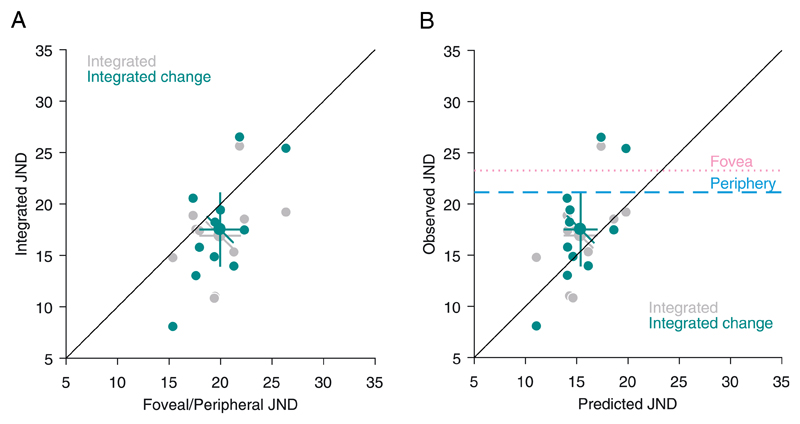
Optimality of integration (Experiment 4). (A) Just-noticeable differences (JNDs) in the integration condition as a function of the best single condition (peripheral or foveal) for every participant. Only three participants in the integration condition without change and two in the integration condition with color change were worse in the integration condition than in their best single condition (above the solid line). (B) Comparison of predicted JNDs with observed JNDs in each integration condition. For color change as well as for no change during a saccade, mean integration performance was close to the optimality line. The dotted line indicates the mean JND value for of the foveal condition, and the dashed line marks the mean JND of the peripheral condition.

Where PSEs and JNDs proved to be relatively similar for both integration conditions—JNDs of integrated versus integrated with change, *t*(10) = −0.47, *p* = 0.648—the pattern of best-of-single-conditions JNDs versus integration-condition JNDs (Figure 7A) also appears to be alike for no change and color change. Participants were better in integration conditions than in the best single condition: best of single versus integrated, *t*(10) = 2.42, *p* = 0.018 (one-sided); best of single versus integrated with change, *t*(10) = 2.08, *p* = 0.032 (one-sided). A comparison of the JNDs predicted from the single conditions (15.37 ± 2.47) with the observed JNDs in both integration conditions revealed that, again, performance was slightly worse than predicted (Figure 7B) but close to optimality: observed versus predicted without change, *t*(10) = −1.39, *p* = 0.196; observed versus predicted with color change, *t*(10) = −1.76, *p* = 0.109.

In our last experiment, PSE values among the single and integration conditions revealed a similar pattern as we found in our first experiment. This and the reduced JNDs for both integration conditions compared to the single conditions indicate that participants integrated numerosity information across saccades even with the 100%/0% black-and-white ratio and color change between the targets.

### Questionnaire

In the second, third, and fourth experiments, numerosity stimuli could change during a saccade. To evaluate whether participants consciously perceived such a change, they were asked to fill in a questionnaire after each experiment and say whether they perceived a change within the numerosity stimulus during a trial. In Experiment 2, none of the 13 participants reported such a change. In Experiment 3, eight out of 13 participants perceived a change within a trial. In Experiment 4, 10 of the 12 participants reported having seen a color change.

## Discussion

Near-optimal integration of pre- and postsaccadic information has been shown previously for low-level stimuli such as spatial orientation (Ganmor et al., 2015; Wolf & Schütz, 2015). Here we show that near-optimal integration of peripheral and foveal input can be achieved as well for a high-level visual feature: numerosity. In Experiments 1 and 4, the integrated percept was more accurate despite different biases of peripheral and foveal perception. Furthermore, the integrated percept was more precise than the peripheral and foveal percepts alone and only slightly worse than the one predicted by maximum-likelihood estimation. Experiments 2, 3, and 4 showed in addition that local and global changes in low-level stimulus properties, such as the location of individual dots in the dot field or the color of the whole dot field, did not impair transsaccadic integration.

### Calibration and integration of perceived numerosity across the visual field

Optimal transsaccadic integration of numerosity is challenging, since numerosity perception differs significantly in the parts of the visual field (Valsecchi et al., 2013; Anobile et al., 2015). For example, Valsecchi et al. (2013) have shown that numerosity is underestimated in the periphery when a peripheral stimulus is directly compared to a stimulus in the fovea. In contrast, in our Experiments 1 and 4 we found an underestimation of numerosity in the fovea and an overestimation of numerosity in the periphery. The differing directions of effects in these studies suggest that the misestimations of numerosity might depend on properties of the stimuli and experimental procedure. Nevertheless, we could show that a highly reliable and accurate integrated percept emerged despite significant differences in foveal and peripheral perception of numerosity.

The most apparent difference between the study by Valsecchi et al. (2013) and our study is certainly the constraints on eye movements: Participants were continuously fixating in the study by Valsecchi et al., whereas our participants had to execute saccades to the stimuli. As suggested by Valsecchi et al. and by Anobile et al. (2015), visual crowding might be the source of underestimating numerosity in the periphery. In turn, work by Harrison, Mattingley, and Remington (2013) has shown that the preparation of a saccade can reduce or even abolish visual crowding for the targeted stimulus. Since all our trials involved an eye movement, it is likely that visual crowding was reduced for stimuli in the periphery, which might reduce or attenuate the underestimation of numerosity in the periphery. Another factor leading to rather accurate peripheral perception might be transsaccadic recalibration (Herwig & Schneider, 2014; Valsecchi & Gegenfurtner, 2016). For example Herwig and Schneider (2014) have demonstrated that peripheral perception is biased toward a postsaccadic, foveal percept after sufficient exposure to this sequence. The interleaved integration trials within our paradigm might be sufficient to induce such transsaccadic associations. Thus, the peripheral percept might be biased toward the foveal percept expected after a saccade. Finally, the small but significant underestimation we found in the fovea might be due to the size or potentially asymmetrical shape of the attention window (Cutzu & Tsotsos, 2003; Stewart & Ma-Wyatt, 2017). Since presaccadic target stimuli in the foveal trials were small fixation crosses, the attention window might have been rather small in order to match the size of the target stimulus present at the time (Ghahghaei & Verghese, 2017). According to Cutzu and Tsotsos (2003), there is an annulus of inhibition directly surrounding the attended location. The foveal target duration might have not been sufficient for the attention window to adapt to this substantially larger numerosity stimulus present after the saccade. This small, inhibited area might have cut off a small part of the relatively large numerosity stimulus such that numerosity was perceived to be lower in foveal trials.

Importantly, the integrated percept seems to have balanced out the biases of the foveal and peripheral percepts, which led to an accurate estimate. This is in line with the assumption of the maximum-likelihood estimation model that an integrated percept should lie in between the percepts of the components. Moreover, the integrated percept should be more inclined toward the more reliable percept, which would intuitively be the foveal percept in this case. Our data do not meet this prediction, since there is a slightly higher weighting on the peripheral input for PSE values than is predicted by means of the JND values. Other factors, such as reduced crowding in the periphery or small attention windows in foveal trials, might have influenced the reliability of each percept. Furthermore, small differences in the presentation duration of the foveal stimulus might have led to an overestimation of the predicted foveal weight.

Generally, one could discuss whether participants based their perceptual judgments on numerosity or on texture density (for a review, see Anobile et al., 2016). Since we did not randomize potential cues like dot size or the size of the circular area, we cannot rule out the possibility that participants relied on texture density (Gebuis & Reynvoet, 2011, 2012). Given recent findings (Anobile et al., 2013; Cicchini et al., 2016; Zimmermann & Fink, 2016), it seems that numerosity is used for small numbers and sparse stimuli, while density is used for large numbers and dense stimuli. Estimates for the transition between numerosity and density mechanisms range between 0.25 dots/°^2^ (Anobile et al., 2013) and 2 dots/°^2^ (Cicchini et al., 2016). The densities for the stimuli we used ranged between 0.98 and 3.91 dots/°^2^. Given these magnitudes, it is assumable that judgments could rely on both numerosity and texture density. However, independent of which cues participants might have used here, these cues were integrated across saccades. If, as suggested by Anobile et al. (2015), foveal information is judged by numerosity but peripheral information by texture density due to crowding, it is even more interesting that such distinct modality judgments (Anobile et al., 2013) can be integrated almost optimally and lead to a more reliable judgment on numerosity than one of them alone. The same applies for Experiments 2, 3, and 4, where the brightness of the stimulus could also have been used as a cue.

### Disrupting object continuity

Theoretically, transsaccadic integration could occur on a low-level, image-based representation (transsaccadic fusion) or on a high-level, abstract representation (transsaccadic memory). According to fusion theory, pre- and postsaccadic stimuli would be fused mandatorily into one percept. This overlay implies that a change of color from black to white and vice versa should result in at least partly gray-colored dots. Since the background was also gray for the numerosity stimuli, fewer dots should have been perceived in the fused percept. In general, our results showed very little influence of stimulus changes on the accuracy of numerosity judgments, suggesting that numerosity information has been extracted from the pre- and postsaccadic stimuli separately before integration takes place.

The fact that performance was not impaired by the color change or even position change of the dots might speak for a summary-statistics mechanism being involved (Saiki & Holcombe, 2012). Summary statistics are referred to as mechanisms serving for perception given a rich input but a limited computational capacity (Attarha, 2015). These mechanisms are thought of as extracting the underlying statistics of the environmental input by finding statistical regularities among items of similar kind. For example, the task of all four experiments of this study involves summary statistics through extracting the mean of the numerosities presented. The information of the mean over all given stimuli should reach awareness to be of use; however, summary statistics are also assumed to occur in early visual processing. In the special case of visual presentation in the periphery, the brain is assumed to pool information in an area which increases its size with eccentricity (Balas, Nakano, & Rosenholtz, 2009). This pooling discards information about individual objects in a scene but extracts useful information on the ensemble. Since we found no difference in performance in most of the conditions, such a higher level mechanism is likely to apply for numerosity estimation. If there were a low-level mechanism at work—for example, every dot is assigned to a single neuron— integration performance should have gotten worse with color or position change.

Our results furthermore show that near-optimal integration across saccades is possible despite disruptions in object continuity. This is interesting because several studies have reported that the perception of differences between pre- and postsaccadic information is facilitated by blanking the target (Deubel, Schneider, & Bridgeman, 1996; Weiß, Schneider, & Herwig, 2015) or by changing target features (Poth, 2015; Poth & Schneider, 2016). Recently, transsaccadic perception of position has been modeled in a causal inference framework (Atsma, Maij, Koppen, Irwin, & Medendorp, 2016), in which pre- and postsaccadic position signals are integrated or segregated depending on the probability that they come from the same or a different source. In contrast, our findings indicate that integration can be achieved despite clear changes in other, unrelated object features. Therefore, the decision between integration and segregation seems to be more flexible and might be modulated by demands and goals of the current task set.

### Neural basis

Finally, our results might help to uncover the neural basis of transsaccadic integration. One potential mechanism supporting transsaccadic integration is predictive remapping (for reviews, see Melcher & Colby, 2008; Higgins & Rayner, 2015), a phenomenon where neurons show presaccadic activity in response to visual stimuli that will be in their receptive field only after the saccade (Duhamel, Colby, & Goldberg, 1992). Predictive remapping is considered an important feature of the brain to gain perceptual stability across eye movements (for a review, see Hall & Colby, 2011). Neurons with predictive remapping were first identified in the lateral intraparietal area (Duhamel et al., 1992) and are also present in several visual areas (Nakamura & Colby, 2002; Merriam, Genovese, & Colby, 2007). However, they seem to be more prevalent in higher areas of visual processing such as V3 and V4 than in lower processing areas such as V1 and V2. Further evidence for a crucial contribution of parietal cortex comes from a study documenting impairments in transsaccadic memory due to transcranial magnetic stimulation over parietal cortex (Prime, Vesia, & Crawford, 2008). Interestingly, the parietal cortex (Harvey, Klein, Petridou, & Dumoulin, 2013)—especially the lateral intraparietal area (Roitman, Brannon, & Platt, 2007)—is also involved in the processing of number (for reviews, see Nieder & Dehaene, 2009; Piazza & Izard, 2009). Our finding that the transsaccadic integration of numerosity was not affected by changes in low-level features matches nicely with the encoding of numerosity in parietal cortex that shows a higher prevalence of remapping responses than early visual areas. Robust estimation of numerosity despite differences in stimulus properties is also a hallmark of the number sense (Nieder & Miller, 2004), indicating that numerosity can be perceived irrespective of the transient disruptions in visual processing caused by saccadic eye movements.

## Conclusion

This study shows that transsaccadic information integration is possible for complex features such as numerosity. The benefit of transsaccadic integration in precision appears to remain even when object continuity is disrupted. This identifies transsaccadic integration as a highly robust mechanism that helps the visual system to create a stable perception of our environment. Numerosity perception per se becomes more accurate with the integration of peripheral and foveal numerosity information compared to one of the inputs alone. This stresses the assumption that transsaccadic integration not only maximizes information gain but also alleviates miscalibrations of peripheral and foveal vision to maintain a stable perception of our environment.

## Footnotes

1 With this paradigm, we cannot make assertions about the actually perceived numerosity, only about the momentary relation of the stimuli and the percept. For instance, if a participant consistently overestimated every stimulus seen (e.g., perceived numerosities of 22, 32, 42, 46, 52, 58, 62, 72, and 82 instead of 20, 30, 40, 44, 50, 56, 60, 70, and 80), he or she would still show a PSE close to 50 (the mean numerosity would still be rated as the PSE).
